# What do computer scientists tweet? Analyzing the link-sharing practice on Twitter

**DOI:** 10.1371/journal.pone.0179630

**Published:** 2017-06-21

**Authors:** Marco Schmitt, Robert Jäschke

**Affiliations:** 1 Institute of Sociology, RWTH Aachen University, Aachen, Germany; 2 Information School, University of Sheffield, Sheffield, United Kingdom; 3 L3S Research Center, Hannover, Germany; Institut Català de Paleoecologia Humana i Evolució Social (IPHES), SPAIN

## Abstract

Twitter communication has permeated every sphere of society. To highlight and share small pieces of information with possibly vast audiences or small circles of the interested has some value in almost any aspect of social life. But what is the value exactly for a scientific field? We perform a comprehensive study of computer scientists using Twitter and their tweeting behavior concerning the sharing of web links. Discerning the domains, hosts and individual web pages being tweeted and the differences between computer scientists and a Twitter sample enables us to look in depth at the Twitter-based information sharing practices of a scientific community. Additionally, we aim at providing a deeper understanding of the role and impact of altmetrics in computer science and give a glance at the publications mentioned on Twitter that are most relevant for the computer science community. Our results show a link sharing culture that concentrates more heavily on public and professional quality information than the Twitter sample does. The results also show a broad variety in linked sources and especially in linked publications with some publications clearly related to community-specific interests of computer scientists, while others with a strong relation to attention mechanisms in social media. This refers to the observation that Twitter is a hybrid form of social media between an information service and a social network service. Overall the computer scientists’ style of usage seems to be more on the information-oriented side and to some degree also on professional usage. Therefore, altmetrics are of considerable use in analyzing computer science.

## 1 Introduction

Twitter communication has permeated every sphere of society [1]. To highlight and share small pieces of information with possibly vast audiences or small circles of the interested has some value in almost any aspect of social life. As such, Twitter is also used by researchers [2] and it is regarded as a means to assess the importance and impact of scholarly articles by measuring how often their links are shared on Twitter. Yet, to date a comprehensive analysis of which content is actually shared by researchers on Twitter does not exist. The value of Twitter for academic fields or for researchers is therefore unclear, while the trend of a “maturation of the platform” [3, 4] and its evolution from a conversation-based social network to an information-sharing-based news media platform indicates a growing importance of professional usage. Therefore, in this work we will focus on the sharing of URLs by the community of researchers in the field of computer science to evaluate the role of Twitter in academic communication.

Communication plays a central role in the epistemic cultures [5] of science and entire fields of research on science are devoted to analyze the patterns in scholarly communication [6]. There is a clear understanding that scientific disciplines publish and communicate differently and that they use different channels and adapt to new communication technologies in particular ways [7, 8]. Styles of communication (especially in new communication environments like social media) [9–11] can be a major focus for their differentiation.

Computer science seems to be an especially advanced field in appropriating web-based communication technologies into their communication strategy, either because they work on such technologies themselves or because there is a heightened affinity to the usage of computer-mediated communication. This is reflected in the restructuring of the field with the upcoming subject of human-computer interaction [12, 13]. This field has grown bigger and bigger in computer science and has strong and reinforcing relations with a couple of other subject areas like computational graphics, software engineering, web technologies, data management and information retrieval. This transformation makes computer science a very interesting testbed for the evaluation of information sharing practices of scientists on Twitter as part of Science 2.0 [14, 15].

Existing analyses of researchers using Twitter are often limited to tweeting behavior during conferences [16–30] or sharing of journal articles. [31–33]. In particular, they are not really aimed at analyzing a preferably complete population of researchers from a specific discipline, in part due to the difficulty of collecting a reasonable sample of researchers.

Ross, Terras, Warwick, and Welsh [23] describe Twitter as an ideal medium to establish a “more participatory conference culture”, since it can expand communication and participation. Consequently, over the last few years there have been several analyses of Twitter usage during academic conferences [16–30]. There is also a major interest in the relationship between scholarly publications and their citations on Twitter [31–36] with mixed results and diverging databases. Another concern are the disciplinary differences in Twitter usage [37]. Overall, the studies show that Twitter is relevant for researchers, also beyond conferences [23, 34, 38], which indicates the need for holistic analyses of how researchers use Twitter. Such kind of analyses have not been done yet, because researchers were restricted to identifying academic users through surveys and interviews or through conference hashtags. The huge interest in such kind of analyses is therefore hampered by the lack of simple methods to identify researchers that are using Twitter and discerning what they are tweeting about. Additionally, there is a lack of understanding on the overall demographics of Twitter users and a lack of key demographic indicators [39]. Moreover, there are likely other biases in Twitter data as well because of over- and under-representation of certain population groups [40]. For instance, in a study based on Twitter users in the UK, Sloan et al. find an over-representation of young people (median 15-25 years) and the creative sector [41]. Overall, the identification of demographic properties of Twitter users is challenging due to their heterogeneity. Existing approaches mostly focus on sub-populations, for instance, users of a specific country [40], and are therefore not well-suited for a comprehensive study of a diverse and large group of users. The identification problem can be tackled in a multidimensional way, by combining the Twitter following of major conferences and literary databases [42]. More generally, different approaches for identifying “research content” on Twitter exist [25]: identifying tweets that contain research content (which [43] consider very difficult) or links to scholarly content [44], identifying users that are researchers [34, 42], or identifying scientific hashtags [21, 27].

Overall, there is also a broad discussion on the identity of Twitter as a social network site or an information site [45, 46]. A survey by Nature indicates that scientists are prone to use it professionally more as an information medium, but in a broad variety of ways [47]. Tackling these questions on a comprehensive common database will enable us to answer some of them in a more thorough way.

The diffusion of URLs and scholarly papers on Twitter is a major topic in the discussion of altmetrics [48, 49]. The main question revolves around the relationship between classical measures of scientific impact and measures based on social media [50] and how similar they are [51]. What kind of impact has a service like Twitter on scientific communication inside the group of peers and to the broader public? Generally speaking, the discussion of altmetrics is focused on the question of the relevance of altmetrics and therefore social media for scholarly communication [32]. These kind of questions relate to another important topic concerning Twitter, which relates to how tweets, hashtags and links gain popularity on Twitter. Important findings show support for a two-step communication flow, with a high impact of prominent “elite users” and a large population of intermediaries [52] and that top rank influencers are characterized by network-related measures [53]. There is also the notion that user categories for elite users (like celebrities, media, organizations, and blogs) have a major influence on what is retweeted [52]. Finally, there is an ongoing discussion on the advantage of Open Access for citations [54, 55] which converged on a moderate advantage that can also impact sharability on Twitter. A recent study on the impact of Academia.edu on citations [56] shows evidence that online platforms may have a citation advantage via discoverability that may pertain to Twitter as well. Altmetrics derived from different sources might reflect different types of impact. For instance, while Mendeley might be an indicator for academic impact, altmetrics on Twitter might reflect impact in the broader public [33, 36]. Furthermore, the coverage of publications depends on the system: for a given set of publications, dedicated systems like Mendeley or CiteULike have a much higher coverage than other services like Facebook or Twitter [49]. We expect a differing relevance for different fields and would like to start a discussion on this topic related on our findings for the field of computer science. With respect to altmetrics or article-level metrics, current approaches which leverage tweet counts do not distinguish between different types of users (e.g., researchers vs. non-researchers or experts from different scientific disciplines).

The overarching research question we tackle in this work is: What type of content do computer scientists share on Twitter? Specifically, we seek to answer the following research questions:

1. What are the TLDs, hosts, publishers and specific URLs computer scientists shared most in 2014?
2. Can we identify a specific usage style on Twitter by computer scientists for the sharing of web links?
3. What kind of scholarly publications are shared the most and which are the publications to be shared specifically by computer scientists in 2014?
4. Are there indicators for a widespread professional use of Twitter by computer scientists on Twitter?
5. What are the implications for the discussion of altmetrics and their value for computer science?

Tackling these questions depends on a comprehensive database that includes all relevant researchers that are using Twitter for a given field, like the computer scientists in our study. Identifying such communities on Twitter is hard, because of duplicates, ambiguous names, and pseudonyms.

We perform a comprehensive study of computer scientists using Twitter and their tweeting behavior. Specifically, we focus on the URLs shared in tweets, since they are readily available and enable an easy aggregation (e.g., on the domain or host level) and partitioning (e.g., by using existing lists of URLs). Answering the research questions mainly became possible due to the availability of three large datasets:

- A collection of almost 9,200 Twitter accounts of computer scientists [42] enables the analysis of a large set (several million) of tweets from researchers.
- The provision of data from the Microsoft Academic Graph [57] enables the identification of scholarly content using URLs.
- The continuous collection of the Twitter 1% sample stream by our institute enables a comparison of the researchers’ tweets against the tweets of the general Twitter population and therefore the identification of content specifically relevant for computer scientists.

The novel combination of those three datasets enables insights which were not possible before at that scale. To answer the research questions we are specifically analyzing

1. the measures which are appropriate to determine the importance of URLs,
2. the differences which exist between computer scientists and the general Twitter population, in particular,
3. the content providers and publications that are specific to researchers.

We expect differences in areas of special interest information (like relevant blogs), with regard to scientific publishers and repositories. Answering these questions will also enable us to give an account of the relevance of altmetrics for studying computer science. Our approach to focus on tweets from researchers enables a peer-review like consideration of content relevance and to distinguish between impact in the (wider) academic community and the general public. Specifically, our approach can be leveraged for the recommendation of tweets and scholarly content. The data underlying this analysis is available at DOI: 10.5281/zenodo.580587.

This paper is organized as follows: In Section 2 we describe the datasets, pre-processing procedure, and analysis methods we have used. In Section 3 we present the results and in Section 4 we conclude with a discussion and an outlook on future work.

## 2 Materials and methods

Our approach requires a comprehensive collection of datasets and pre-processing which we describe here. We then explain the methods we applied to answer our research questions.

### 2.1 Datasets

The analysis is based on three datasets. We analyze an existing dataset about computer scientists on Twitter and compare the tweeting behavior against a sample of the Twitter stream. Furthermore, we leverage data from the Microsoft Academic Graph to classify the content of tweets.

#### 2.2 Twitter data about computer scientists

Our analysis is based on data about computer scientists using Twitter [42, 58]. The dataset was collected in January 2014 based on a seed list of 170 Twitter accounts of computer science conferences. All Twitter users that retweeted, followed, or were followed by one of those seed accounts were collected. The resulting list of 52,678 candidates was then matched with 73% accuracy against the computer science bibliography DBLP [59] by comparing the real names from the Twitter accounts with the author names from DBLP [42]. The resulting 9,191 matched candidate users are the starting point for this analysis. We collected all tweets of these users that they have posted during the year 2014, which resulted in 2,259,756 tweets from 6,694 users. We then removed all tweets which did not contain at least one URL (we extracted URLs from the expanded_url field of the Twitter APIs JSON data). The resulting collection comprises 957,480 tweets (42%) by 6,271 users (94%).

#### 2.1.2 Twitter sample

Since access to the complete Twitter stream is expensive, we used the 1% sample stream that is provided by Twitter (https://dev.twitter.com/streaming/reference/get/statuses/sample) to create a sample collection of tweets. We are aware of the fact that this is not a perfect sample [60, 61] but given the lack of alternatives we consider it the best available data source to perform our comparison. We focus our analysis on frequent items (e.g., URLs that were tweeted by many users) and mainly aggregations of URLs (e.g., hosts, domains), since the 1% sample preserves the frequency of important domains in URLs very well [61]. We collected all tweets from the 1% stream for the year 2014 which resulted in 1,605,361,179 tweets from 117,907,194 users. Again, we removed all tweets without at least one URL. The resulting collection comprises 300,053,850 tweets (19%) by 51,072,970 users (43%).

We also used the 1% sample stream to collect a random sample of 6,694 users that had tweeted in 2013 and also had at least one tweet in 2014. As in the computer scientists dataset, we collected all tweets of those users from 2014 which resulted in 2,966,723 tweets. We then removed all tweets which did not contain at least one URL, resulting in a collection of 591,875 tweets (20%) by 5,646 users (84%). We used this dataset for a user-based comparison of the computer scientists’ data with the sample.

#### 2.1.3 Microsoft Academic Graph

The Microsoft Academic Graph (MAG) data [57] contains metadata for 118,693,328 scholarly publications from 102,444,355 authors, including 275,049,539 URLs where the papers or their metadata have been published. We computed a list of the 10,000 most frequent host names among this set of URLs and used this list to identify links to scholarly content within tweets. We restricted our analysis to the top 10,000 hosts (of 239,212), since we are interested in the most important sources where researchers can find published works. Furthermore, the dataset is quite noisy and contains a long tail of sites such as imdb.com, newyorker.com, or youtube.com beyond the top 10,000 which clearly do not host scholarly publications.

### 2.2 Pre-processing

To enable the comparison of URLs from the different datasets, we had to solve two problems: expansion and normalization.

#### 2.2.1 Expansion

Many URLs that are posted on Twitter are *shortened* by so-called URL shortening services. The result is that different short URLs can refer to the same (long) URL. Therefore, these short URLs need to be identified and expanded—typically by sending an HTTP request and then extracting the original URL from the response’s “Location” header field. URLs that were shortened by Twitter’s integrated shortening service are returned in their original form by the API. However, many URLs are shortened by other services, e.g., 10.6% of the URLs in the Twitter sample point to the popular service Bitly. To expand the URLs in the researcher data, we used a list of 340 shortening services from http://longurl.org/services to identify shortened URLs and expanded them by using the described method. Unfortunately, the large amount of shortened URLs in the sample data (around 74 million or 44%) prevented us from expanding the URLs in this dataset. Therefore, we did not expand the URLs in the sample dataset (except for URLs which are also contained in the researcher data) and assume that the distribution of URLs on the shortened subset is similar to the distribution on the not-shortened subset. Since we are mainly analyzing the URLs in aggregations on the host and domain level, our assumption is even weaker, namely that hosts and domains are similarly distributed over both sets.

We removed all shortened URLs which we could identify using the aforementioned host list from the sample dataset. For the shortening services youtu.be and fb.me we made an exception, since they link exclusively to YouTube and Facebook, respectively, and are very popular: we changed the host names of the corresponding URLs to youtube.com and facebook.com, respectively. The alternative of removing URLs with those two host names would have considerably disturbed the top ranking.

This cleansing procedure reduced the number of users from around 51 million to 45 million. Nevertheless, all fractional values we report for this dataset were computed with the 51 million users. This step mostly affects the analysis on the host, domain, and TLD level (i.e., Sections 3.3 and 3.4). In Section C in S1 Appendix we show a comparison on the TLD level to see which influence this cleansing step has.

#### 2.2.2 Normalization

The same web page can be represented by many different URL strings. We included several common steps (like transforming the host name to lower case, collapsing the path, etc.) using the Python package urlnorm. In addition, we also sorted the query parameters alphabetically and kept the fragment (i.e., the part after the hash sign ‘#’), since we identified some services which deliver different content depending on the value of the fragment and removing it collapsed many URLs into one URL.

In general, even with normalization it is difficult to automatically decide whether two URLs refer to the same content. The effort for crawling the content and comparing it is huge and the benefit is questionable, since comparing the content raises new problems—it could have changed or is no longer available, or the same content could have different representations. Therefore, we used this basic URL normalization and did not apply more sophisticated methods.

One exception is the list of top publications in Section 3.6 where we applied a semi-automatic normalization procedure. For each publication in the top list (and 10 publications beyond), we manually extracted the publication id from the corresponding URL and searched for other URLs containing this id. We then checked those newly discovered URLs and replaced URLs which also referred to the publication by the original URL. One example which shows where this is necessary is provided in Table 1. All four URLs refer to the same article and have quite different numbers of users. Another common example are URLs which have tracking parameters (e.g., from Google Analytics) attached such that many unique URLs exist which point to the same content. We are aware that our focus on only the top publications might miss some publications who are spread over several URLs which did not make it into the top list (in the worst case, each user tweeted an individual URL). However, we found only few variations (mostly for the same publishers like PLOS and Nature which have different types of URLs) and we observed that popular publications are frequently retweeted. The retweets preserve the URL such that typically one URL for each such publication is among the top URLs.

**Table 1:** Four example URLs which all refer to the same publication.

#users	URL
13	http://dx.plos.org/10.1371/journal.pone.0115069
14	http://journals.plos.org/plosone/article?id=10.1371%2Fjournal.pone.0115069
21	http://www.plosone.org/article/info:doi/10.1371/journal.pone.0115069
13	http://www.plosone.org/article/info:doi%2F10.1371%2Fjournal.pone.0115069

*An Efficiency Comparison of Document Preparation Systems Used in Academic Research and Development* by Knauff and Nejasmic, 2014

### 2.3 Methods

**Fig 1:**
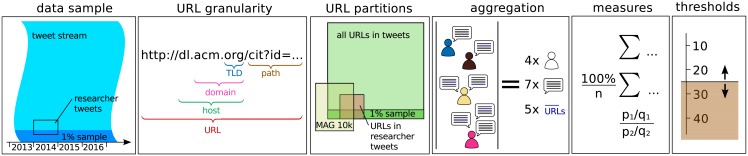
Overview on the different aspects of the analysis.

To analyze the content that is shared by computer scientists on Twitter, we mainly focus on the URLs they share in their tweets and the publications these URLs refer to. Given the richness of the data, there are several options on how to select, aggregate, and analyze it. The different options are visualized in Fig 1 and described in detail in this section.

#### 2.3.1 Data sample

As described in Section 2.1 we restrict our analysis to tweets from 2014 from the Twitter 1% sample stream and from the computer scientists data set. We choose the year 2014, since for that year we had collected the complete 1% sample stream. Each analysis can be performed on one of the datasets but we will also compare both datasets with each other.

#### 2.3.2 URL granularity

We can decompose a URL like http://adsabs.harvard.edu/abs/2012arXiv1203.4745P into its constituent parts: the host name adsabs.harvard.edu, the (second-level) domain harvard.edu, and the top-level domain (TLD) edu and then perform aggregation and counting on the different parts. In general, the different parts of the host name are separated by dots and thus can be extracted by splitting the host name at the dots. However, some top-level domains like uk or jp have a layer of second-level domains to separate different types of hosts, e.g., ac.uk for academia, co.uk for commerce, etc. Typically, the second-level domain can be used to identify companies and institutions which would not work for these cases. To have a comparable granularity for the second-level domains independent of their top-level domain, we therefore need to handle such cases. Since there is no generic way to extract such second-level domains, we use readily available lists, for instance, https://en.wikipedia.org/wiki/Second-level_domain. This enables us to extract “second-level” domains like nii.ac.jp from URLs such as http://research.nii.ac.jp/ntcir/evia2014/.

#### 2.3.3 URL partitions

We analyze all URLs contained in the tweets of the two datasets. To further differentiate these URLs and to identify URLs which point to scholarly content, we partition them using the Microsoft Academic Graph data. This partitioning can be performed on the URL, domain, and host level. On the URL level this would mean that only URLs from tweets which appear in the MAG data are considered as “publisher URLs”. This is quite restrictive, since relevant URLs could have been missed in the MAG collection process for several reasons (e.g., URLs in the tweets could be newer than the MAG data). Therefore, partitioning on the host or domain level is more appropriate. Partitioning URLs on the domain level would certainly include host names that are not contained in the MAG data. For instance, the host names academicpositions.harvard.edu and adsabs.harvard.edu would be treated the same, despite the fact that the first host name does not appear in the MAG list. Partitioning on the host name level could fix this issue but would ignore the common case where only the domain (which often is also used as a host name) appears in the MAG list. For example, the host name www.vldb.org would be ignored since only the host/domain name vldb.org is contained in the MAG list. To solve these issues, we are using a suffix match, that is, URLs are considered to be “publisher URLs”, if their host name or a suffix of their host name is contained in the list of the 10,000 most frequent MAG hosts. This solves the aforementioned problems and allows us to partition URLs into “publisher URLs” and “other URLs” and later on to identify relevant publications.

#### 2.3.4 Aggregation

We can identify popular items (e.g., URLs or domains) by counting in how many *tweets* the items are contained or by how many *users* they have been tweeted. Counting tweets provides a simple measure of popularity but it can also be biased by users promoting their content. For instance, a user posting a tweet for every new blog post can easily cause the host name of the blog to appear among the top hosts in the data. Another example is the service Paper.li which automatically posts tweets with a URL on a user’s account, when the corresponding page has been updated. Therefore, we also count for each item the number of distinct users that have shared it. Consequently, users posting several tweets with different URLs to the same host or domain are counted just once for that host or domain, respectively.

In this aggregation step, we also have to consider the temporal aspect that younger URLs (or tweets) had less time to get (re)tweeted. In our analysis we are focusing on data from the year 2014 and thus content that was created at the very end of the year 2014 is likely less popular just because it had not enough time to gain enough (re)tweets. Since popular hashtags exhibit a burst time period of at most a few days [62], we assume a similar behavior for URLs and therefore expect that only few tweets and URLs from the very last days of the year could be affected.

#### 2.3.5 Measures

We are using both raw tweet and user counts and normalized (percentage) counts (fractions). Although the counts give a good overview on the popularity of the items they do not allow us to identify items that are especially popular for computer scientists but less popular among the overall Twitter population represented by the sample. To compare these two sets of users, we apply the odds ratio [63] by setting the counts for the computer scientists in relation to the counts in the sample and then ranking the items by their odds ratio. This also allows us to identify items that are less popular among computer scientists. More specifically, we compute the odds ratio for an item *i* based on the number of users that tweeted this item as follows:

$$\begin{array}{c} OR\left(i\right)=\frac{|U_{CS}\left(i\right)|}{|U_{CS}\backslash U_{CS}\left(i\right)|}\cdot \frac{|U_{S}\backslash U_{S}\left(i\right)|}{|U_{S}\left(i\right)|} \end{array}$$

where *U*_*CS*_ (*U*_*S*_) represents all computer scientists (sample users) and *U*_*CS*_(*i*) (*U*_*S*_(*i*)) represents the set of users in the computer scientists (sample) dataset that have shared item *i*. Thus, the odds ratio is the ratio between the odds of finding a computer scientist that has shared the item versus the odds of finding a “regular” user that has shared the item. The higher the ratio, the more specific an item is for the computer scientists. Similarly, we can also compute the odds ratio based on the number of tweets.

For comparison of rankings we use the Spearman correlation coefficient *ρ* since it is well suited for heavy-tailed data [64] as it is found on Twitter. We use a random rank between 0 and 1 for items that do not appear in a ranking to avoid ties.

#### 2.3.6 Thresholds

To focus on the relevant parts of the data, we introduce thresholds that ignore items that have been shared by few users or in few tweets only. This also avoids overestimating the importance of infrequent items [61]. We motivate the choice of thresholds for the odds ratio computation in Section 3.4. In addition, we manually remove outliers from some datasets. We explicitly discuss these cases. For instance, when we rank hosts by their odds ratio between the number of users that have shared them in the computer scientists data versus the number of users that have shared them in the 1% sample, we ignore hosts that have been shared by only few users in the sample, since the sample does not contain enough evidence. All reported correlations and confidence intervals have a significance level of *p* < 0.001.

## 3 Results

After a comparison of the basic characteristics of the two groups of Twitter users, we analyze the difference in counting tweets vs. counting users. We then focus on three aspects relevant to our research question: the domains relevant for computer scientists, the difference when considering the relative importance compared to the overall Twitter population, and the URLs and publications that are specifically relevant for computer scientists.

### 3.1 Basic characteristics

We present basic statistics about the three tweet collections in Table 2. As mentioned before, the analysis is focused on the tweets which contain a URL (“URL tweets” by “URL users”). For comparison, in this section we also report some statistics for all tweets and all users of the corresponding datasets. For the sample data we include both tweet collections, since—as we will see—the sampling based on users yields different characteristics than the sampling based on tweets. Since the data exhibits a long tail of URLs that appear in only one tweet or have been tweeted by only one user, the median for the corresponding counts is 1 in all datasets. From the table we can estimate that around 30 billion tweets were posted in 2014 which contain a URL (i.e., 100 times as much as we observe in the 1% sample). The 51 million users in our dataset cover a large fraction of the overall Twitter population in 2014 which is around a fifth of the 241 million monthly active users in 2014 [65].

**Table 2:** Basic statistics of the three tweet collections.

measure		computer scientists	sample users	sample tweets
number of	users	6,694	6,694	117,907,194
	URL users	6,271	5,646	51,072,970
	tweets	2,259,756	2,966,723	1,605,361,179
	URL tweets	957,480	591,875	300,053,850
	tweet-URL pairs	989,529	605,080	319,879,232
	URLs (raw)	796,474	454,111	169,124,498
	URLs (expanded)	766,611	–	169,124,498
	URLs (normalized)	762,918	453,648	168,881,714
$\mu \pm \sigma \;(\tilde{x})$	tweets per user	337.6±630.8 (72)	443.2±609.1 (175)	13.6±2435.6 (2)
	URL tweets per URL user	152.7±301.6 (40)	104.8±234.9 (26)	5.9±36.2 (2)
	tweets per URL	1.3±3.1 (1)	1.3±22.8 (1)	1.9±496.9 (1)
	users per URL	1.2±1.1 (1)	1.0±0.7 (1)	1.5±143.2 (1)
	URLs per user	144.8±285.0 (38)	83.4±197.6 (20)	5.0±30.7 (2)

The lower part shows the mean (*μ*), standard deviation (*σ*), and median ($\tilde{x}$).

As can be seen, there is a big difference in the user-based statistics between the collection based on the random sampling of *tweets* versus the random sampling of *users*. This is expected, since the random tweet sample does not contain complete tweet histories for users and therefore the mean and median number of tweets and URLs per user are much lower. To gain an insight into the difference in the tweeting behavior between computer scientists and “regular” users, we therefore included the statistics for the equally-sized random sample of users which contains all of their tweets. For the following user-based comparison we will use this dataset. There is a big difference observable from the outset between sample users and computer scientists in that the scientists are much more prone to include links in their tweets. While the mean and median number of tweets per user are much higher for the sample users than for the computer scientists, computer scientists are more likely to share URLs in their tweets. There are 104.8 mean URL tweets per user with a URL in the sample but computer scientists have 152.7 mean URL tweets per URL user. Because we just look at tweets with URLs this discrepancy is unusually high and stays roughly the same for the mean URLs per user (144.8 to 83.4). This implies that computer scientists use Twitter in a special way to communicate many links to spread important information. This big difference in usage should be kept in mind for the entirety of our analysis. In addition, we have to keep in mind that there are demographic differences between the users in the sample and the computer scientists. For instance, the computer scientists are older on average and better educated, which may lead to quite different behavior on Twitter.

Another observation is that for both collections the number of tweet-URL pairs is higher than the number of tweets. This means that some tweets contain several URLs and one could expect that therefore the mean number of URLs per user is higher than the mean number of tweets per user. However, as Table 2 shows, this is not the case. An explanation is that users have tweeted the same URL several times which results in a lower mean number of URLs per user than tweets per user.

**Fig 2:**
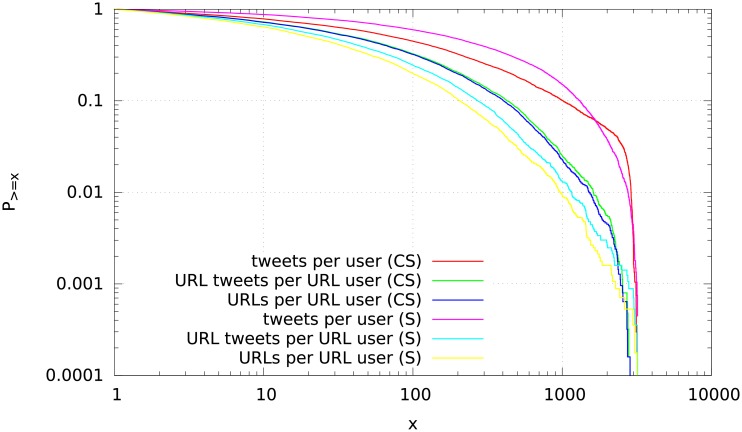
The complementary cumulative distribution functions of the number of tweets per user and the number of URLs per user during 2014 for the URL tweets of the 6,271 computer scientists (CS) and the 5,646 sample users (S). The “tweets per user” curves show the corresponding distributions for *all* tweets of the 6,694 computer scientists and sample users, respectively.

Taking a closer look at the distribution of the number of URL tweets and URLs per URL user (Fig 2) we can see that in general the distributions are similar in both datasets, although the number of tweets with a URL is smaller (591,875) in the sample data than in the researcher data (957,480). Overall, there is a larger fraction of computer scientists with medium to high numbers of URL tweets and URLs. The “tweets per user” distributions for all tweets are almost converse: there is a larger fraction of sample users with a medium number of tweets and a smaller fraction with a high number of tweets compared to the computer scientists. Overall, the plot confirms the differences observed in Table 2 but also indicates that the focus on URL tweets yields similar changes in both datasets.

**Fig 3:**
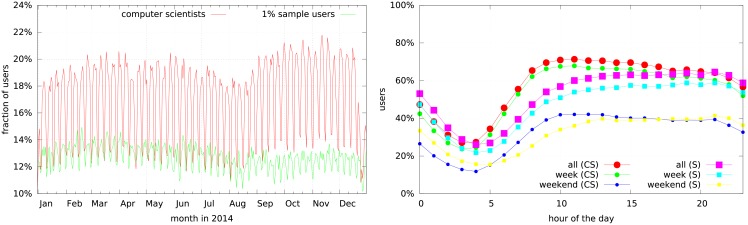
The percentage of users that was active during a specific day of the year (left) and a specific hour of the day (right) for the computer scientists (CS) and sample (S) datasets. The times were normalized by regarding the time zones of the users from their Twitter profile, if they were available (around 60% of all users have a time zone set in both datasets), else the users were ignored.

Having a look at the timing of the activity on Twitter (cf. Fig 3) suggests that the bulk of activity of computer scientists is happening over workdays and working hours. Though, in general, the curves are very similar (all Pearson correlations of the corresponding curves are larger than 0.69 with *p*-values smaller than 0.001), we can see a clear difference in both plots between computer scientists and regular users: computer scientists are more active and there is a larger difference in the number of users that are tweeting between workdays and weekends. While both datasets show a lower tweet volume during August, the highest activity for the computer scientists is in October and November, while for the regular users it is in February and March. Furthermore, the difference of the number of tweeting users between day and night is more pronounced for the computer scientists. Their minimum is at around 3 to 4 AM which is followed by a steep ascent to a maximum at 11 AM and then a slow descent. The ascent in the sample data is less steep and reaches its maximum at 9 PM. Although the level of activity is different for both datasets during weekdays and weekends, the progression of the corresponding curves is similar. Overall, the increased activity on workdays and over working hours is another indication of a semi-professional use of Twitter by computer scientists. The temporal pattern when considering all tweets (not just tweets with URLs) is very similar, as discussed in Section A in S1 Appendix.

### 3.2 Differences in counting tweets, users, and URLs

For our comparison of the information sharing behavior of computer scientists and “regular” Twitter users we focus on the URLs they have frequently shared and the resulting domains and top-level domains (TLDs). To decide which ranking (based on the number of users, tweets, or URLs) is most suitable, we first compare the three rankings for the domains of the URLs that were shared by the computer scientists. We find that the Spearman rank correlation between the rankings by the number of URLs and by the number of tweets is higher (*ρ* = 0.91) than between the rankings by the number of users and tweets (*ρ* = 0.86) and users and URLs (*ρ* = 0.79). For an in-depth analysis, we compare the top 20 rankings for the computer scientists dataset based on the number of tweets, users, and URLs per domain, respectively (cf. Table A in S1 Appendix which shows the corresponding rankings, including the counts and fractions). In the top 20, there is a clear difference between counting the number of tweets or the number of users or URLs. Among the top domains by the number of tweets are domains of services for automatically posting content on Twitter. The services swarmapp.com, paper.li, and scoop.it have a tendency to produce many tweets per user (on average 28.7, 40.8, and 21.1, respectively). For instance, tweets containing a URL to paper.li frequently announce changes on the corresponding paper.li sites of the Twitter users by posting the URL to this site. These occur every day, because new news reports are introduced. Consequently, paper.li has a very high tweets per URL ratio of 15.8. For all other sites among the top 20 this ratio is close to 1 which in turn means their tweets per user and URLs per user ratios are very similar. Therefore, the rankings by the number of URLs and by the number of tweets are very similar, as indicated by their high Spearman correlation (*ρ* = 0.91). Since the counts based on the number of users are more stable and less susceptible to high-volume tweeting of individuals, for all subsequent analyses we will use these counts.

### 3.3 Relevance of TLDs and domains

We focus on top-level domains (TLDs) and domains first, because they enable the most general outlook on what is important for a specific Twitter community, like the computer scientists. The TLDs rely on the general categorical topography of the domain name system, also underlying the world wide web. Spheres of society like countries (uk or de) or domains (org or edu) are therefore observable which play an important role in the Twitter communication of a certain community.

The user-based rankings of the TLDs for the computer scientists data and the sample data are highly correlated (Spearman’s *ρ* = 0.79, *p* < 0.001). On the TLD level such a high correlation can be expected, since some TLDs (e.g., com, org) are generally much more frequent than other TLDs (e.g., mobi) and therefore TLDs exhibit a similar distribution in both datasets. Considering the ranking of the top 15 TLDs for the computer scientists in Table 3, we can observe a general tendency for a higher relevance of organizational and education-related TLDs for the computer scientists. The TLD org is considerably higher in the ranking and edu, gov, eu and ac.uk are also at relatively high positions. The TLD io can be considered as particularly relevant for computer scientists since “io” is commonly associated with “input/output” and therefore several technology-oriented sites use the domain. Taking the corresponding top 20 ranking for the sample data into account (cf. Table B in S1 Appendix) we observe that some TLDs commonly used by URL shortening services like ly, be and gl lose influence in the computer scientists data, because they have been expanded. There are also some differences among the country TLDs. For instance, the TLD of Germany, de, appears in the top list of the computer scientists, while Japan’s jp is not among the top 20. (One reason for the absence of jp is that it got split into ac.jp, co.jp, etc.) The ranking of the TLDs for computer scientists also suggests a stronger emphasis on sharing information of general or public interest, as well as for information that is scientifically relevant. We will see further evidence for that when we analyze the links in a more detailed way.

**Table 3:** The top 15 TLDs for the computer scientists dataset.

	TLD	#users	%users
1	com	5,938	94.69%
2	org	4,399	70.15%
3	net	3,401	54.23%
4	edu ▲	2,515	40.11%
5	co.uk	2,326	37.09%
6	co	1,980	31.57%
7	io ▲	1,924	30.68%
8	de ▲	1,718	27.40%
9	ly	1,603	25.56%
10	me	1,528	24.37%
11	gov ▲	1,431	22.82%
12	it	1,369	21.83%
13	ca ▲	1,140	18.18%
14	eu ▲	1,134	18.08%
15	ac.uk ▲	1,122	17.89%

The TLDs are ordered by the number of users (#users) which have posted a URL with the corresponding TLD in one of their tweets. The highlighted TLDs (▲) do not appear among the top 20 of the sample.

Coming down on actual domains, we first observe that the sample contains a much larger number of distinct domains (2,199,027) than the computer scientists dataset (84,235). Naturally, the user counts on the domains are not correlated (Spearman’s *ρ* = 0.02, *p* < 0.001), since only 61,836 domains appear in both datasets and the remaining domains are ranked randomly after the existing domains. On the intersection of the domains, the Spearman correlation is larger (*ρ* = 0.36, *p* < 0.001) but considerably lower than on the TLDs. This indicates a distinctive difference in the overall information sharing behavior between computer scientists and general Twitter users.

Among the top 20 domains (cf. Table 4) it appears that links to videos have a special place on Twitter with high rankings for YouTube (ranks 1 and 2), the video sharing service Vine (rank 4 in the sample data), and Vimeo for the computer scientists (rank 16). Links to social networks are relevant, too. Whereas Facebook holds the higher position in the sample data (rank 3 compared to 5 for the computer scientists), Twitter itself seems more important for the computer scientists (rank 3 compared to 7 in the sample). Twitter-related services (like unfollowers.com, uapp.ly, fllwrs.com) do not appear in the top list for the computer scientists and services for photo sharing (Instagram holds position 1 for the sample and 11 for the computer scientists) have higher relevance for the general Twitter population then for computer scientists. Blogs (WordPress, Blogspot), technology-oriented domains (GitHub, Wired, TechCrunch), knowledge-sharing domains (Slideshare, Wikipedia) and news media domains (New York Times, Guardian, Medium, Wall Street Journal, Washington Post) are all in the top list for the computer scientists only. All this points to a much more information-oriented and to some degree professional usage of Twitter compared to the general Twitter population. Different types of information are relevant for the sharing process. Therefore, we can identify different pertinent styles of information sharing, where computer scientists share less private information then the average Twitter user, but much more information of public and general or peer-group-specific (scholarly) relevance. Therefore, Twitter can be regarded as a good place to find content relevant for an academic community.

**Table 4:** The top 20 domains for the computer scientists dataset and for the sample, ordered by the number of users.

	computer scientists			sample		
	domain	#users	%users	domain	#users	%users
1	youtube.com	3,741	59.66%	instagram.com	8,268,717	16.19%
2	google.com	2,390	38.11%	youtube.com	6,930,076	13.57%
3	twitter.com	2,164	34.51%	facebook.com	5,243,498	10.27%
4	wordpress.com	1,970	31.41%	vine.co	4,910,634	9.61%
5	facebook.com	1,941	30.95%	twitter.com	3,289,243	6.44%
6	nytimes.com	1,931	30.79%	ask.fm	1,672,538	3.27%
7	github.com	1,710	27.27%	unfollowers.com	1,207,399	2.36%
8	wired.com	1,652	26.34%	uapp.ly	963,553	1.89%
9	theguardian.com	1,626	25.93%	path.com	825,788	1.62%
10	tumblr.com	1,619	25.82%	edu.sa	633,249	1.24%
11	instagram.com	1,527	24.35%	fllwrs.com	613,842	1.20%
12	medium.com	1,486	23.70%	moi.st	608,207	1.19%
13	slideshare.net	1,407	22.44%	twitpic.com	558,392	1.09%
14	techcrunch.com	1,365	21.77%	soundcloud.com	505,597	0.99%
15	blogspot.com	1,358	21.66%	twcm.me	486,662	0.95%
16	vimeo.com	1,342	21.40%	tumblr.com	485,911	0.95%
17	wikipedia.org	1,326	21.14%	swarmapp.com	409,063	0.80%
18	wsj.com	1,147	18.29%	po.st	406,380	0.80%
19	washingtonpost.com	1,126	17.96%	blogspot.com	403,017	0.79%
20	github.io	1,104	17.60%	justunfollow.com	399,428	0.78%

Another observation is the difference of the distribution of the domains over the users: while all domains among the top 20 of the computer scientists have been shared by at least 10% of the users, only the top three domains from the sample stream have been shared by more than 10% of the users. This can be explained by the fact that the sample data does not contain complete user time lines, that is, all tweets of the users. Another explanation could be the more public-oriented communication style of the computer scientists, compared to a more personal-oriented style in the sample data.

### 3.4 Relative importance

Up to now we have compared rankings based on the absolute numbers of users. Although we could identify some differences between the rankings for the computer scientists and the general Twitter population, the rankings also bear some similarities. We are now using the odds ratio to identify content that is specifically relevant for computer scientists. The odds ratios are based on the user counts. Depending on the URL granularity and partition, we introduce different thresholds to anticipate noise, missing (due to sampling) and sparse information. On the domain and host level (Table 5) we use a threshold of 10, that is, only domains and hosts that have been shared by more than 10 users in the sample are included. For publisher URLs and domains (Tables 6 and 7), we use a lower threshold of 5, since these items are much less frequent in the sample data (e.g., only 10 publisher URLs have been shared by more than 20 users in the sample). Since we could not find similar prior analyses we could build upon, we had to choose the thresholds based on our experience with the dataset. Our goal was to find a balance between identifying relevant items and loosing information such that we can focus on items for which enough evidence in the sample dataset is available. The values for the 99.9% confidence intervals for the odds ratios are provided in Section D in S1 Appendix. In addition, the full rankings are provided in the accompanying dataset (DOI: 10.5281/zenodo.580587).

**Table 5:** The top 20 domains and hosts from the computer scientists dataset, ordered by the odds ratio.

	domain	OR	#u_CS_	#u_S_	host	OR	#u_CS_	#u_S_
1	lemire.me	52,647	108	17	yahoolabs.tumblr.com	79,062	170	18
2	videolectures.net	51,518	75	12	dl.acm.org	56,710	410	63
3	computer.org	44,520	165	31	lemire.me	52,647	108	17
4	johndcook.com	40,682	108	22	videolectures.net	50,899	68	11
5	acm.org	40,306	1,023	247	cacm.acm.org	48,131	325	58
6	socialmediacollective.org	38,117	74	16	www.computer.org	46,650	140	25
7	regehr.org	32,279	55	14	www.johndcook.com	44,750	108	20
8	yhathq.com	31,785	58	15	stanford.edu	40,658	74	15
9	scikit-learn.org	31,355	61	16	nlp.stanford.edu	39,805	82	17
10	strataconf.com	29,893	94	26	socialmediacollective.org	38,117	74	16
11	datasociety.net	29,667	47	13	www.cs.cmu.edu	37,094	207	47
12	academictorrents.com	29,243	71	20	colah.github.io	32,807	44	11
13	insidehpc.com	28,827	70	20	agenda.weforum.org	32,493	71	18
14	pyimagesearch.com	27,322	40	12	blog.regehr.org	31,687	54	14
15	the-paper-trail.org	26,842	62	19	scikit-learn.org	31,355	61	16
16	usenix.org	26,520	226	72	homepages.inf.ed.ac.uk	31,095	53	14
17	toronto.edu	26,095	54	17	cs.stanford.edu	30,872	119	32
18	might.net	24,669	60	20	homes.cs.washington.edu	30,679	56	15
19	continuum.io	24,598	42	14	strataconf.com	29,893	94	26
20	epsrc.ac.uk	24,586	39	13	www.datasociety.net	29,667	47	13

The odds ratio (OR) is based on the user counts – #u_CS_ and #u_S_ for the computer scientists and the sample, respectively. Only domains and hosts that have been tweeted by more than ten users in the sample are included. The 99.9% confidence intervals for the odds ratio are given in Tables C and D in S1 Appendix.

**Table 6:** The top 20 publisher domains (by the number of users) for both the computer scientists dataset and the sample dataset.

	computer scientists				sample			odds ratio ranking	
	domain	#users	%users	%tweets	domain	#users	%users	domain	OR
1	google.com	2,390	38.11%	1.36%	google.com	313,530	0.61%	ceur-ws.org	156,199
2	acm.org ▲	1,023	16.31%	0.32%	google.com.tr ▲	29,325	0.06%	aaai.org	71,015
3	nature.com	817	13.03%	0.26%	google.ca ▲	9,500	0.02%	nott.ac.uk	65,657
4	mit.edu	765	12.20%	0.17%	nasa.gov	7,451	0.01%	umontreal.ca	56,202
5	arxiv.org ▲	677	10.80%	0.34%	abc.net.au ▲	7,103	0.01%	umd.edu	53,475
6	stanford.edu	663	10.57%	0.15%	google.co.jp ▲	5,846	0.01%	vldb.org	47,775
7	microsoft.com ▲	561	8.95%	0.11%	google.de ▲	4,455	0.01%	computer.org	44,520
8	sciencemag.org	396	6.31%	0.09%	nature.com	4,272	0.01%	arizona.edu	42,967
9	springer.com ▲	337	5.37%	0.06%	nih.gov	4,153	0.01%	acm.org	40,306
10	sciencedirect.com ▲	319	5.09%	0.06%	europa.eu	2,522	0.00%	aclweb.org	40,221
11	europa.eu	291	4.64%	0.11%	xataka.com ▲	2,145	0.00%	gla.ac.uk	35,831
12	cmu.edu ▲	282	4.50%	0.04%	bmj.com ▲	1,925	0.00%	ucsb.edu	35,439
13	doi.org ▲	249	3.97%	0.06%	google.com.br ▲	1,905	0.00%	utah.edu	35,072
14	wiley.com	247	3.94%	0.04%	google.co.uk ▲	1,865	0.00%	toronto.edu	35,061
15	usenix.org ▲	226	3.60%	0.05%	sciencemag.org	1,783	0.00%	cmu.edu	32,943
16	nih.gov	208	3.32%	0.05%	wiley.com	1,693	0.00%	tue.nl	31,550
17	nasa.gov	205	3.27%	0.04%	google.as ▲	1,687	0.00%	soton.ac.uk	30,688
18	pnas.org ▲	194	3.09%	0.04%	stanford.edu	1,499	0.00%	cornell.edu	30,148
19	plos.org ▲	188	3.00%	0.04%	cdc.gov ▲	1,443	0.00%	ucdavis.edu	29,203
20	plosone.org ▲	182	2.90%	0.03%	mit.edu	1,386	0.00%	sigcomm.org	28,601
	**any**	3,807	60.71%	5.34%	**any**	452,270	0.89%		

Domains in the computer scientists’ top 20 that do not appear among the sample top 20 have been highlighted (▲) (and vice versa). The %tweets column shows the fraction of tweets in the computer scientists datasets that contain a URL with the corresponding publisher domain. The ranking by the odds ratio (OR) only includes domains that have been shared by more than five users in the sample dataset. The last row shows the counts for any publisher domain, e.g., the number of users who have posted a tweet with a URL from a publisher domain.

**Table 7:** The top publications from the computer scientists dataset.

	publication	year	#cit	#u_CS_	OR
1	◇ Repeatability and benefaction in computer systems research. *Collberg, Proebsting, Warren*	2014	5	69	94,702
2	△ Genes mirror geography within Europe. *Novembre et al.*	2008	720	45	36,914
3	□ Publishers withdraw more than 120 gibberish papers. *van Noorden*	2014	44	**118**	36,276
4	◇ Python is now the most popular introductory teaching language at top U.S. universities. *Guo*	2014	19	76	32,977
5	◇ Interactive notebooks: Sharing the code. *Shen*	2014	29	28	32,723
6	◇ Deep neural networks are easily fooled: High confidence predictions for unrecognizable images. *Nguyen, Yosinski, Clune*	2014	98	45	30,762
7	◇ Please put OpenSSL out of its misery. *Kamp*	2014	4	26	26,579
8	□ An efficiency comparison of document preparation systems used in academic research and development. *Knauff, Nejasmic*	2014	1	57	24,657
9	◇ The network is reliable. *Bailis, Kingsbury*	2014	16	17	23,138
10	□ Publishing: The peer-review scam. *Ferguson, Marcus, Oransky*	2014	36	24	21,802
11	△ Rotational splittings with CoRoT, expected number of detections and measurement accuracy. *Goupil, Lochard, Samadi, Barban, Dupret, Baglin*	2006	1	28	20,824
12	○ Links that speak: The global language network and its association with global fame. *Ronen, Goncalves, Hu, Vespignani, Pinker, Hidalgo*	2014	27	24	17,838
13	◇ To wash it all away. *Mickens*	2014	0	20	16,341
14	□ The missing piece to changing the university culture. *Schillebeeckx, Maricque, Lewis*	2013	29	25	15,725
15	□ Scientific method: Statistical errors. *Nuzzo*	2014	170	**96**	13,690
16	○ Experimental evidence of massive-scale emotional contagion through social networks. *Kramer, Guillory, Hancock*	2014	422	**45**	13,184
17	□ Lectures aren’t just boring, they’re ineffective, too, study finds. *Bajak*	2014	4	26	12,508
18	□ The rise and rise of citation analysis. *Meho*	2007	227	12	12,240
19	◇ An updated performance comparison of virtual machines and linux containers. *Felter, Ferreira, Rajamony, Rubio*	2014	67	9	12,234
20	○ Trolls just want to have fun. *Buckels, Trapnell, Paulhus*	2014	89	9	12,234
a	□ Online collaboration: Scientists and the social network. *van Noorden*	2014	85	**79**	9,309
b	△ Variation in melanism and female preference in proximate but ecologically distinct environments. *Culumber et al.*	2014	3	**73**	9,548
c	□ Nature promotes read-only sharing by subscribers. *van Noorden*	2014	2	**63**	9,255
d	◇ Deep learning. *Bengio, Goodfellow, Courville*	2014	71	47	128,558
e	○ Big data, hype, the media and other provocative words to put in a title. *Jordan*	2014	0	44	90,220
f	◇ First-person hyper-lapse videos. *Kopf, Cohen, Szeliski*	2014	37	**43**	8,816
g	◇ Computer science: The learning machines. *Jones*	2014	0	40	81,966
h	□ How to build a bad research center. *Patterson*	2014	0	40	∞
i	◇ Do we need hundreds of classifiers to solve real world classification problems?. *Fernández-Delgado et al*.	2014	152	35	143,325
j	□ The top 100 papers. *van Noorden, Maher, Nuzzo*	2014	72	**32**	10,392
k	◇ Extracting audio from visual information. *Hardesty*	2014	1	**32**	7,080

The top 20 publications are ranked by their odds ratio (OR). Only publications that were shared by more than five users in the sample are included. Ten publications of the top 20 by the odds ratio are also among the top 21 (there is a tie) when sorting by the number of users (#users). These publications were shared by more than 27 computer scientists. The remaining eleven publications (a–k) are shown in the lower part of the table. Among those are five publications (d, e, g, h, i) with a high odds ratio—they are not among the top 20 by the odds ratio since they were shared by less than six users in the sample dataset. The other six publications were shared by at least 26 users in the sample dataset. The nine publications which have a bold number of users, were shared by more than 20 users in the sample. The number of citations (#cit) is from Google Scholar as of March 3, 2016. The top list also contained some non-publications, which we have removed, but we retained news articles, e.g., from ACM Queue. The symbols encode the following topics: ◇ computer science, □ general academic interest, ○ interdisciplinary, △ other.

Table 5 shows the domains and host names that exhibit the strongest differences according to the odds ratio. We can see a clear pattern of links to domains and hosts which are of special value and importance for computer scientists. The kind of value (be it a specific interest for an area of research or more general academic value) differs a lot.

There is some overlap between domains and hosts (videolectures.net, socialmediacollective.org, scikit-learn.org, strataconf.com), because second-level domains are often also used as host names, but the overall impression according to the odds ratio is a very specific focus on relevant themes for computer scientists. While Yahoo Research on Tumblr gets the top rank and Microsoft’s Research Blog (socialmediacollective.org) is placed on the 10th position, we have some active blogs from computer scientists and professional programmers as well (lemire.me, johndcook.com, blog.regehr.org, colah.github.io refer also to blogs), some hosts of academic institutions with a strong position in computer science (stanford.edu, cs.cmu.edu, nlp.stanford.edu, cs.stanford.edu, homepages.inf.ed.ac.uk, homes.cs.washington.edu), the ACM has two hosts in the top 20 (dl.acm.org and cacm.acm.org) and some sites with general or project related information for computer scientists. We can see that video lectures play some role in Twitter-based communication of computer scientists, with the domain videolectures.net ranked second and the host by the same name at rank four. This site is dominantly used for lectures in computer science with 11,182 lectures and the social sciences as a distanced second with 2,095 lectures.

As can be seen, the host stanford.edu is among the top 20 but not the domain, although the odds ratio of the host (40,658) would be sufficient for the domain to be among the top 20 as well. This can be explained by the fact that several hosts from stanford.edu are more popular among the users of the sample than among the computer scientists. For instance, the host www.gsb.stanford.edu of the Stanford Graduate School of Business was tweeted by 264 users in the sample but only by 23 researchers. The host stanford.edu on the other hand, is frequently tweeted by researchers but only seldom in the sample. This can also be observed for videolectures.net, where the counts for the domain are larger, since they comprise more host names.

### 3.5 Links to publishers

Having observed a remarkable prevalence of domains and hosts relevant for computer science (and thus an indication for the professional use of Twitter) we now shift our focus to clearly work-related content: scholarly publications. Therefore, we analyze the distribution of publisher URLs within the two datasets (cf. Table 6), that is, URLs we identified using the approach described in Section 2.3.3.

Overall, 3,807 (61%) of the computer scientists have posted a tweet with a URL of a publisher domain. In the sample dataset, this is the case for less than 1% of the users. Since the sample dataset does not contain complete user time lines, this is an underestimate of the real share of publisher domains. We therefore counted on the 5,646 sample users for which we had the complete time lines and which had posted a tweet with a URL how many of the URLs in their tweets point to a publisher domain. Overall, 313 of the 5,646 users (5.54%) had tweeted a link to a publisher domain. Though this is more than on the complete sample data, it is dominated by links to hosts from google.com: 471 of the 735 distinct URLs (64.08%) point to google.com—a larger share than on the researcher data (10,141 of the 34,960 distinct URLs (29.01%)). Omitting URLs to google.com in both datasets, we see a clear difference: a majority of 3,351 of the 6,271 computer scientists (53.44%) have tweeted a link to a publisher domain (excluding google.com) but only 115 of the 5,646 users from the sample (2.04%) have done so, too. A comparison on the tweet level shows that 0.14% of the sample tweets contain a URL with a publisher domain, whereas 5.34% of the tweets from the computer scientists have that property, which is almost forty times as much. The two user-based rankings of publisher domains are only weakly correlated (Spearman’s *ρ* = 0.36, *p* < 0.001), indicating that computer scientists favor different publishers than the sample users.

Google seems to be a vitally important publisher, but this is caused by the large collection of patents it hosts at https://www.google.com/patents/ which is contained in the MAG data. The tweets of the computer scientists are usually not pointing to the patents but overwhelmingly to Google Docs, Sites or Google+. Google is all over the place in the sample, with many regional domains in addition to google.com. We include them here, but google.com should not count as a typical publisher domain. The same goes for nasa.gov, which is included in the MAG 10,000, but gets tweets predominantly for its TV channel. The most dominant classic academic publisher on Twitter seems to be nature.com with a share of 13.03% (3rd) of the computer scientists and also ranked 8th in the sample. Other publishers on both lists are nih.gov (16/9), europa.eu (11/10), wiley.com (14/16), stanford.edu (6/18), mit.edu (4/20), and sciencemag.org (8/15). These are the publishers that share a wider audience than computer scientists or scientists in general. There is a score of important publishers missing from the sample, like arxiv.org (ranked 5th in the computer scientists’ data with a user share of 10.8%), springer.com (9th), sciencedirect.com (10th), usenix.org (15th), doi.org (13th), plos.org and plosone.org (19th & 20th), pnas.org (18th). There is just one domain that ranks high overall for computer scientists and according to the odds ratio: acm.org (2/9). ACM ist obviously especially important for computer scientists. Overall the user shares by the computer scientists run from 38.11% or 2,390 users (google.com) to 2.90% or 182 users (plosone.org). Interestingly, stanford.edu and mit.edu have a relatively broad appeal to general users as well, while there are a lot of educational and academic institutions with an appeal to computer scientists in the ranking based on the odds ratio. There are organizations relevant for computer scientists on the one hand, e.g., ceur-ws.org (1st), aaai.org (2nd), vldb.org (6th), computer.org (7th), acm.org (9th), aclweb.org (10th), and sigcomm.org (20th), and universities on the other hand. This is an interesting list, because these institutions are especially interesting for tweeting computer scientists. They are notts.ac.uk (3rd), umontreal.ca (4th), umd.edu (5th), arizona.edu (8th), gla.ac.uk (b11th), usbc.edu (12th), utah.edu (13th), toronto.edu (14th), cmu.edu (15th), tue.nl (16th), soton.ac.uk (17th), cornell.edu (18th) and, ucdavis.edu (19th)—all universities with established computer science departments.

**Fig 4:**
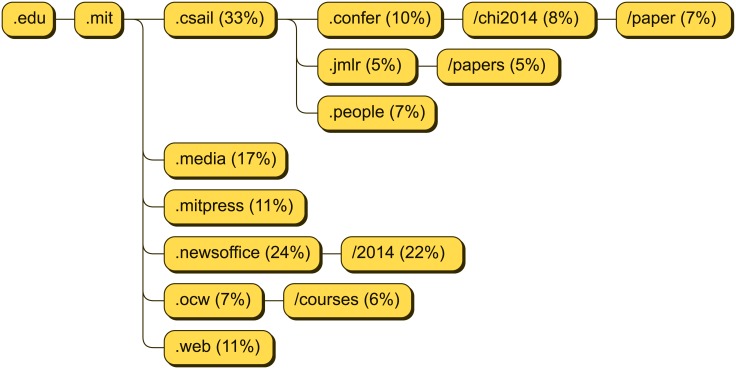
A visualization of the frequent subdomains, host names, and paths for the domain mit.edu. All subdomains and paths which were contained in URLs that were tweeted by at least 5% of the computer scientists that had tweeted a URL to mit.edu are shown. The numbers give the corresponding percentages of users.

The spread of links containing a specific domain is usually quite large. As an example we show the actual frequencies for the domain mit.edu within the computer scientists dataset (Fig 4). We see that about one third of all links to MIT shared over Twitter by computer scientists are directed to CSAIL (csail.mit.edu), MIT’s renowned Computer Science and Artificial Intelligence Laboratory, and another 17% to the MIT Media Lab (media.mit.edu), both departments that are especially interesting for researchers in computer science and which host publications, journals, and conference sites. Also, a considerable share goes to MIT Press (mitpress.mit.edu) for publication links and to MIT’s news from 2014 (http://newsoffice.mit.edu/2014), for general information on what was happening at MIT. Obviously, most of the tweeted links that point to mit.edu are of considerable professional interest to computer scientists.

### 3.6 Relevant URLs and publications

While the preceding analyses focused on higher-level patterns of information sharing among computer scientists we now reduce the granularity and directly analyze the most frequently shared URLs. This enables us to identify topics and publications that are relevant for computer scientists.

We focus on URLs pointing to scholarly publications by restricting the set of URLs to those whose host names do appear among the top 10,000 MAG hosts. (For comparison, the converse analysis—URLs whose host names do *not* appear in that set—can be found in Section E in S1 Appendix.) Table 7 shows the top 20 publications, ranked by their odds ratio. Nine of those twenty publications are also among the top 20 when ranking the publications by the number of researchers who have shared them. The remaining eleven publications from that ranking can be seen at the end of the table. The two different rankings allow us to differentiate between publications that are specifically relevant for computer scientists (first 20 publications) and those that are also relevant in general (6 of the last 11 publications).

This list is quite interesting, because it shows that most of the publications prominently positioned in the computer science community on Twitter are actually from 2014, the year of the data set. There are just 4 exceptions: the 2nd ranked article is from 2008, the 11th from 2006, the 14th from 2013, and the 18th from 2007. All of them are not publications from computer scientists. Two of them stem from other disciplines (2 and 11) and two are of general scientific interest (14 and 18). Nonetheless, most publications are from computer science (◇, 14 out of 30), 4 are interdisciplinary and related to computer science (△), 11 are of general scientific interest (□), and 3 are from other disciplines (○). Publications mix special and general scientific themes pretty evenly. Citation counts are very diverse, which is a bit surprising, considering the short time since their publication. Paper no. 16 has over 400 citations in Google Scholar in less than two years, it is an interdisciplinary study, the famous massive emotional contagion experiment on Facebook. Clearly, this reached our sample because it was highly controversial and also of high interest for computer science. Two articles from other disciplines are highly visible inside the computer science Twitter community not because of their scientific value, but for things that do not happen usually in scholarly texts. They have just one or three citations, respectively, but the first (paper no. 11) contains an anti-acknowledgment and the second (paper b) has left a citation remark (“should we cite this crappy paper”) in the final version. The second ranked paper is interesting too, because it is from another discipline (biology/genetics), had significant scientific impact (720 citations), is from 2008 and has no direct connection to computer science. Attention is focused on the visualization part, where a PCA of the genome of 3,000 Europeans reveals a map of Europe. We cannot say why this article resurfaced in the computer science community in 2014, but this boundary spanning events (publications crossing the boundaries between disciplines) are an interesting feature of Twitter for scientists. To identify positions in academic communities on Twitter seems especially interesting in a network-focused approach to our dataset, which we have foreseen as future work (cf. Section 4.4). The same is true for paper no. 18 on the list, which is of general scientific interest, has significant citations (227) and stems from 2007. Most of the tweets about scholarly publications are retweets and this is true for all kinds of publications (see Section F in S1 Appendix for sample tweets). We can show this with a few examples, starting with the more uncommon papers:

- Paper no. 2 on genes mirroring geography has just 3 original tweets inside the community and 42 retweets of these tweets or of tweets from an outside source. They are citing the paper and say how “incredible” or “cool” the results are. All this happens in the short time frame of 3 days.
- Paper no. 11, the one with the “anti-acknowledgment”, has two original tweets from outside the computer science community and one from the inside. The retweets, 29 overall, are spread out over a longer period of time, than in paper no. 2, coming up in April, May, June, October, and November in new but relatively short-lived bursts.
- Paper no. 18, on the rise of citation analysis, is an oddity, because it is relatively old, and has just 12 tweets overall and 9 are retweets from one user outside our sample. The high odds ratio seems to be a coincidence, because we got a late pickup through an outside source in the computer science community.

The more common and highly relevant papers in computer science exhibit some differences compared to these outliers:

- Paper no. 1 on repeatability problems in computer science is from 2014 and has 73 tweets overall (with 14 original and 59 retweets), with a just one major outburst in March and closing pretty soon after (last tweet in May).
- Paper no. d, a new book on deep learning, also from 2014, has 55 tweets (11 original and 44 retweets), shows a similar pattern between tweets and retweets, but shows two major outbursts and a much longer time frame of activity (from June until the end of the year).

Finally, publications of general scientific interest are common too and generate more original activity:

- Paper no. 3 on the withdrawal of 120 fake papers has 120 tweets overall with 54 original and 66 retweets, which points to a lot more original activity and less retweeting. The paper is from 2014 and peaked in March, but generated some activity until the end of the year with another smaller burst of activity between May and June.
- Paper no. a on scientists using social networks has 78 tweets overall with 13 original and 65 retweets, which resemble the specific papers from the field of computer science much more. It is also from 2014 and peaked over a longer time frame from August to September, but did not last as long as paper no. 3.

**Fig 5:**
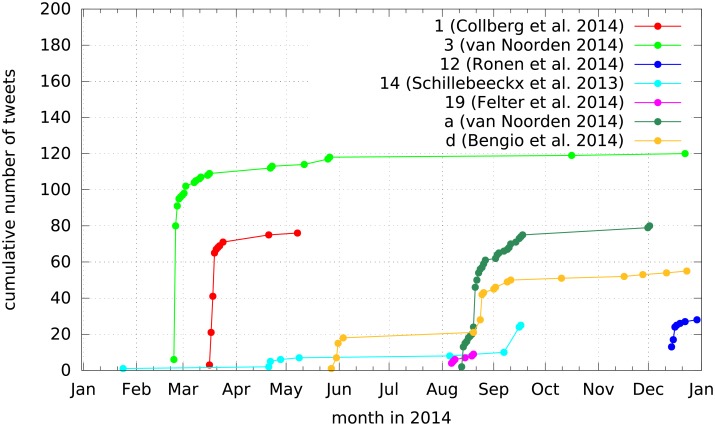
The cumulative number of tweets over the year 2014 for a selection of publications. Each publication is identified by its id in Table 7.

All this is an indication for a hybrid information sharing style on Twitter based on differentiated criteria of relevance for differentiated audiences. To trace the temporal evolution of a selection of publications, we have plotted the cumulative number of tweets in the computer scientists dataset over the year 2014 (cf. Fig 5). In general, we observe typical burst patterns, that is, within a few days many tweets are posted and then the tweet rate drops. This is different for two papers (14 and d) which have two burst time periods within 2014: May and September for paper 14 and June and August for paper d. A further difference is the length of the activity. Paper 1 has a short activity phase (2 months), while these phases are much longer for paper 3 (March till end of year) and paper 14 (8 months).

## 4 Discussion and conclusion

### 4.1 Twitter usage by researchers

The results clearly show that the information shared by computer scientists on Twitter is different from that shared by the general Twitter population. In particular, they share more established news content (e.g., New York Times, Guardian, BBC) and links pointing to resources that are particularly relevant for computer scientists (e.g., GitHub or publisher web sites). The TLDs, domains and hosts that are of particular interest for computer scientists indicate a preference for information- and work-related areas of the web. For instance, the edu and org top-level domains are more likely to be shared by computer scientists, than by the common users of Twitter (see Table 3). The domains are much more likely to include established newspaper sites (New York Times, Guardian, Wall Street Journal, and Washington Post), tech-related sites (GitHub, Wired, TechCrunch) and Blogs (WordPress, Blogspot) (see Table 4). The same is true for the hosts, where institutions like universities and other professional organizations are over-represented (Stanford, Carnegie Mellon, Washington, and ACM), which shows where computer scientists find relevant information via Twitter (see Table 5). This demonstrates the possibility to identify a specific link-sharing style of computer scientists on Twitter, which centers on general public interest and discipline-specific information. While the sharing of news media, especially newspaper sites, could also be explained by an age or education bias of the researcher community, the hybridization between such content of general interest, general academic interest, and close community interest of computer scientists may indicate a researcher style of Twitter usage or a fingerprint of academic usage, as has been observed for unemployment [66]. We also created a top list of publications relevant for the community of computer scientists on Twitter, which shows some of the more general information practices. The top three papers (as well as the whole list) shows the diversity of the relevant information linked on Twitter and includes a computer science paper, a general scientific interest paper and a paper from another discipline (see Table 7). Our results show that the analysis of information sharing practices on Twitter should not be restricted to small datasets or specific events, like academic conferences, because personal styles and event-based patterns can distort overall stylistic regularities like professional usage and the hybridization of audiences and relevance criteria. All these observations underline the relevance of our analysis and are a good justification to perform similar and comparative analyses within and across other disciplines. Additionally, the datasets have much potential for re-usability and the approach should work also for analyzing other disciplines. The computer scientists and their tweets should be further analyzed, because we barely scratched the surface here (see Section 4.4).

### 4.2 Implications for altmetrics

Also, our results have implications for altmetrics. Most striking is the observation that computer scientist indeed frequently use Twitter to post different types of professionally relevant content. This is first indicated on the TLD level (Table 3), where we see that links in the tweets stemming from the TLDs org, net, edu, io, and gov are more frequent than in the sample. The next indication is in Table 4, where we can see that in relation to actual domains, computer scientists share more links to established news sites and information sharing domains than the general Twitter population. There is more emphasis on public information (scholarly or not), while the general Twitter population posts more links to Twitter-specific apps and therefore focuses on personal information and Twitter activities (following and unfollowing). The results on the URL level have mixed implications for altmetrics. Nearly half of the top shared content according to the odds ratio rankings of actual publications (Table 7) is not specific to computer science, but of general scientific or public interest. In addition, the recency of the scholarly publications that were tweeted by the computer scientists underlines an important argument for the value of altmetrics: it can help to identify content early that will gain much attention inside the community. Remarkably, even in the short time span since 2014, the top tweeted publications received a considerable amount of citations. For publications of general relevance for researchers we observed that they are popular among computer scientists as well. What we have discovered here, is mixed or hybrid usage of Twitter to spread information to a hybrid audience. Altmetric analysis of Twitter for computer scientists seems to be particularly valuable to gain an up-to-date overview of relevant themes. This topical relevance can not so readily be reflected by citation analysis, even for a discipline like computer science that is following a fast citation style in communication. For the interpretation of scholarly impact metrics using Twitter, our results suggests a very careful approach. Researchers should try to evaluate their results considering the mixed audiences and relevance criteria in parallel use on Twitter. While there are effects of discoverability on Twitter [56], active researcher communities on Twitter seem to strike a delicate balance between their different audiences on Twitter with a tendency to professional usage. We conclude that a distinct style of information sharing by computer scientists on Twitter can be observed, which is based on sharing quality information with a community-specific focus on the one hand and a broader scholarly and public interest on the other hand. Links to websites with high-quality information for a variety of publics (audiences as diverse as computer scientists, technology developers, scholars in general, and a general media audience) dominate the sharing activities compared to the general Twitter population, which is much more likely to share personal information (like pictures), links to other domains (e.g., religious sites), or even spam. It seems likely that other academic communities share some elements with the style of the computer scientists, like the dominance of quality information sharing and the coverage of narrower and broader publics. On the contrary, personal publics seem to be less relevant for the computer scientists. Though we expect that these narrower publics are aligned with the corresponding scientific disciplines and also cause a change in the style of information sharing. Picking up the discussion of different discipline-specific Twitter practices [37], we can foresee that our more comprehensive sample of computer scientists on Twitter reveals that a very specific style can be identified that reflects the information-heavy link sharing culture for an audience that meshes professional and public interests in computer science and uses Twitter as a kind of news media, adaptable to different audiences.

Computer scientists reside more on the side of professional use, which is one category that was identified in [37], compared to some of the other disciplines mentioned there, which are more privately oriented. This is one dimension where a broader approach comparing the different disciplines might be interesting. Overall, we conclude that Twitter is a good laboratory for altmetrics, because we can clearly discern a professional, information-oriented style of communication, but also observe the hybridization of relevance criteria for different audiences pertinent to Twitter, which should be a concern in altmetric studies. Therefore, the investigation of other disciplines is a consequential next step. While the computer scientists community might be very well adapted for a professional use of online communication networks, other disciplines might cling to a more private style of usage.

### 4.3 (Technical) Lessons learned

The experiments we performed in this work were enabled by the re-use and combination of existing datasets. In particular, the release of the Microsoft Academic Graph data [57] has opened up new opportunities for research. The dataset turned out to be extremely valuable for identifying scholarly content based on URLs at a large scale. We envision many other tasks where it will be useful, for instance, linking Twitter user accounts with the profiles of authors would enable to take their scholarly track record (and thus experience) into account. This could be beneficial for ranking or recommending scholarly content.

Dealing with such a large dataset of content from Twitter raised many challenges. The transience of (shortened) URLs impedes the analysis of the underlying content. The expansion of short URLs is necessary but also difficult due to access rate limitations of the services. We therefore pragmatically decided to ignore shortened URLs on the large sample dataset. Overall, implementing proper URL handling (including normalization, aggregation, comparison, etc.) requires trade-offs. For instance, the fragment part of a URL (everything after ‘##’) is supposed to be handled by the web browser only but some services deliver different content depending on its value. Hence, URLs which are identical except for the fragment not necessarily can be regarded to point to identical content. The selection of appropriate sample data for comparison is straightforward for tweets when one was able to collect the free 1% sample stream, otherwise more elaborate approaches are necessary [27]. Sampling random users is still a challenge, though, since access to user time lines is limited (only the last 3,200 posts can be accessed). Results are different depending on whether analyses were performed on the tweet or on the user level. Some analyses require comparison on the user level which in our case would have restricted the comparison to a much smaller sample. One option to solve this issue is to buy pre-processed datasets from one of the existing data providers, though this is expensive and provides less control over and insight into the different (pre)processing steps which were performed. Another challenge is the categorization of URLs where we used a binary partition (publisher/non-publisher) only. Using a more fine-grained categorization would be preferable but is also challenging. Reliable automatic approaches typically require the web page content which might no longer be available for older URLs and is costly to get and handle for large numbers of URLs. An alternative could be manually classified lists of URLs, like the Open Directory Project (DMOZ, https://www.dmoz.org/). We tested classification of URLs using DMOZ but observed very low coverage, even with the relaxed URL matching process we used for the MAG data. We therefore discarded to analyze the URLs using more fine-grained categories.

### 4.4 Future work

The dataset of computer scientists that are active on Twitter [58] enables a broad variety of further research. Analyzing the temporal aspects of Twitter usage in more depth can help to differentiate between specific periods of more or less activity, identify periodic content sharing, and trends in the mentioned topics. It should be possible to observe how large computer science conferences impact tweeting behavior, how the professional use of Twitter is concentrated in specific time slots, or how the community responds to central events in a given year (e.g., controversial publications or behavior). Another time-related question concerns the tweet-retweet burst patterns of articles or central memes in computer science. Our first explorations suggest some identifiable patterns, which could relate to relevance, network diversity, or polarization.

The second area for future work concerns the internal differentiation of computer science. Which research topics are especially relevant on Twitter and how does this relate to the internal differentiation concerning journals or citation networks in computer science? A related question is how can links be automatically categorized according to their general theme or topic? Such an approach would simplify the differentiation between the structural, temporal, and topical realms of Twitter usage styles.

The third aspect we would like to analyze in more depth, are the links to scholarly publications within tweets and their interpretation. We would like to analyze the connection between networks of tweets/retweets of links to publications and networks of computer scientists to identify central network positions that are related to the bursty temporal patterns of such links on Twitter. The networked dimension of the dataset needs to be further analyzed to grasp the social dimension of the communities within the set of computer scientists on Twitter and to understand how their tweeting style corresponds with their position in the network and their research interests. Therefore, it would be interesting to identify the demographics of the users to take their age, role, country, etc. into account. We also want to analyze the effects of discoverability on Twitter, relating to the possible effects of Open Access policies to information sharing practices. Like the network patterns, this will involve further analysis that is beyond the scope and agenda of this article. Lastly, we would like to compare our findings with other scientific disciplines or other types of professional communities on Twitter to get a better overview on generic and specific styles of Twitter usage for professional and information-intensive communities. We would also like to compare different metrics for assessing the impact of publications derived from Twitter data. Ultimately, we hope that a better understanding of how researchers use social media can help to improve information finding, for instance, by devising novel methods for ranking or recommendation.

## Supporting information

S1 Appendix Additional tables and figures. (PDF)
