# Supplementary material for: What do computer scientists tweet? Analyzing the link-sharing practice on Twitter
Source: PLoS One. 2017 Jun 21;12(6):e0179630. doi: 10.1371/journal.pone.0179630 (PMC5479540; doi:10.1371/journal.pone.0179630)
Supplement: S1 Appendix — (PDF) [file pone.0179630.s001.pdf]

S1 Supporting Information: Appendix

A Temporal Tweeting Activity

Figure A extends the discussion of the temporal tweeting activity in Section 3.1 with a plot that is based on all tweets and not just tweets with URLs. As can be seen, the daily and seasonal patterns are very similar. A noticeable difference is the steady decline of the fraction of users that are active per day in the sample dataset. This is caused by the sampling of users that were active in 2013. Apparently, some of those users stopped using Twitter in the course of 2014. The fact that such a decline can not be observed for the computer scientists could be an indicator for a more sustainable (including professional) use of Twitter, a hypothesis which is worth further investigation. In our initial experiments, sampling of users among those that were active in 2014 caused the opposite effect: a steady increase of activity over the year 2014, since some users became active only at the end of the year.

B Differences in Counting Tweets, Users, and URLs

As discussed in Section 3.2, the analysis is based on user counts of items that appear in tweets. Table A extends the discussion by showing the similarities and differences between rankings based on the number of tweets, users, or URLs.

C Top TLDs

Having a look at the second and third column of Table B, we see that removing URLs from popular URL shortening services has considerably changed the top 20 TLDs of the

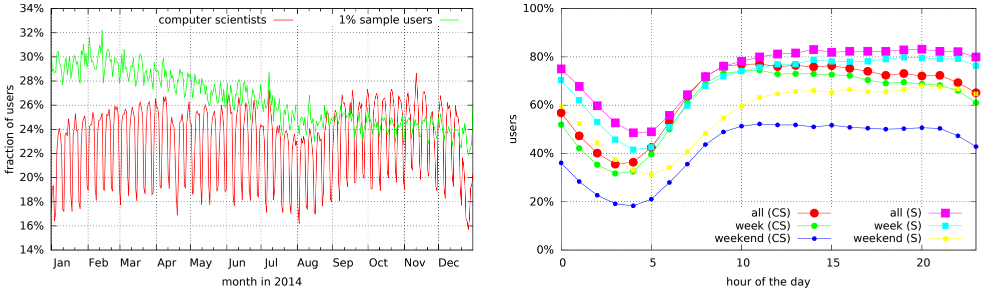

(a) days of the year **(b)** hours of the day

Fig. A. The percentage of users that was active during a specific day of the year (left) and a specific time of the day (right) for all tweets of the computer scientists (CS) and sample (S) datasets. The times were normalized by regarding the time zones of the users from their Twitter profile, if they were available (around 60% of all users have a time zone set in both datasets), else the users were ignored.

Table A. The 20 top domains from the computer scientists dataset, ordered by the number of tweets, users, and URLs, respectively.

	number of tweets			number of users			number of URLs		
	domain	#tweets	%tweets	domain	#users	%users	domain	#URLs	%URLs
1	youtube.com	38,284	4.00%	youtube.com	3,741	59.66%	youtube.com	32,656	4.28%
2	instagram.com	21,851	2.28%	▲google.com	2,390	38.11%	instagram.com	21,658	2.84%
3	facebook.com	17,936	1.87%	▲twitter.com	2,164	34.51%	facebook.com	16,741	2.19%
4	▽swarmapp.com	14,269	1.49%	▲wordpress.com	1,970	31.41%	swarmapp.com	14,247	1.87%
5	google.com	13,033	1.36%	▼facebook.com	1,941	30.95%	google.com	10,114	1.33%
6	github.com	12,520	1.31%	▲nytimes.com	1,931	30.79%	▲nytimes.com	9,406	1.23%
7	nytimes.com	11,843	1.24%	▼github.com	1,710	27.27%	▲twitter.com	9,396	1.23%
8	twitter.com	10,882	1.14%	▲wired.com	1,652	26.34%	▼github.com	8,995	1.18%
9	wordpress.com	10,042	1.05%	▲theguardian.com	1,626	25.93%	wordpress.com	7,326	0.96%
10	▽paper.li	9,667	1.01%	▲tumblr.com	1,619	25.82%	▲tumblr.com	7,084	0.93%
11	theguardian.com	9,123	0.95%	▼instagram.com	1,527	24.35%	theguardian.com	6,723	0.88%
12	tumblr.com	8,529	0.89%	▲medium.com	1,486	23.70%	▲bbc.co.uk	5,700	0.75%
13	▽bbc.co.uk	7,169	0.75%	▲slideshare.net	1,407	22.44%	▲scoop.it	4,921	0.65%
14	medium.com	6,172	0.64%	▲techcrunch.com	1,365	21.77%	▲techcrunch.com	4,653	0.61%
15	techcrunch.com	6,044	0.63%	▲blogspot.com	1,358	21.66%	▲feedly.com	4,428	0.58%
16	slideshare.net	5,772	0.60%	▲vimeo.com	1,342	21.40%	▲wikipedia.org	4,358	0.57%
17	wired.com	5,752	0.60%	▲wikipedia.org	1,326	21.14%	▼slideshare.net	4,068	0.53%
18	blogspot.com	5,157	0.54%	▲wsj.com	1,147	18.29%	▼medium.com	3,863	0.51%
19	▽scoop.it	4,956	0.52%	▲washingtonpost.com	1,126	17.96%	▲vimeo.com	3,861	0.51%
20	wikipedia.org	4,801	0.50%	▲github.io	1,104	17.60%	▼blogspot.com	3,480	0.46%

In the second and third column blocks ▲domains are highlighted that are ranked higher by the number of users or URLs, respectively, than by the number of tweets. Conversely, ▼domains that rank lower in the corresponding ranking than by the number of tweets are also highlighted. The highlighted ▽domains in the "number of tweets" column block do not appear among the top 20 for the "number of users". These are domains for which URLs have been shared frequently but by few computer scientists only.

sample data. For instance, ly (bit.ly), me (fb.me), be (youtu.be), and gl (goo.gl) have lost while other TLDs like net, jp, or org are stable. Nevertheless, the two (complete) rankings are almost perfectly correlated ($\rho = 0.9991, p < 0.001$), since the removal of popular URL shortening services changed the rankings mostly in the top positions.

D Relative Importance

Tables 5, 6, and 7 show rankings based on the odds ratios of items. Tables C, D, E, and F extend the values from those tables by the corresponding 99.9% confidence intervals for the odds ratios. The values show that the lower bounds of all odds ratios are considerably larger than 1, which means that the items are considerably more likely to be shared by computer scientists than by average Twitter users. The intervals also show the large range of possible values, indicating that the rankings can not be seen as measures of absolute importance but rather as a means to identify the most relevant items.

E Top URLs

Table G shows URLs that are specifically relevant for computer scientists (since they have a high odds ratio) but which do not necessarily point to scholarly publications

Table B. The top 20 TLDs for the computer scientists dataset and for the sample dataset

	computer scientists			sample			sample (incl. short URLs)		
	TLD	#users	%users	TLD	#users	%users	TLD	#users	%users
1	com	5,938	94.69%	com	32,351,004	63.34%	com	28,422,779	55.65%
2	org	4,399	70.15%	co	5,477,080	10.72%	ly	9,457,445	18.52%
3	net	3,401	54.23%	net	1,975,637	3.87%	me	6,547,098	12.82%
4	edu ▲	2,515	40.11%	jp	1,842,588	3.61%	co	6,033,097	11.81%
5	co.uk	2,326	37.09%	fm	1,749,259	3.43%	be	4,459,361	8.73%
6	co	1,980	31.57%	org	1,577,175	3.09%	gl	3,136,856	6.14%
7	io ▲	1,924	30.68%	me	1,453,593	2.85%	net	1,981,955	3.88%
8	de ▲	1,718	27.40%	st	1,204,622	2.36%	jp	1,842,589	3.61%
9	ly	1,603	25.56%	ly	1,203,555	2.36%	fm	1,750,186	3.43%
10	me	1,528	24.37%	info	1,180,617	2.31%	org	1,577,196	3.09%
11	gov ▲	1,431	22.82%	es	905,931	1.77%	st	1,282,034	2.51%
12	it	1,369	21.83%	ru	825,209	1.62%	info	1,180,617	2.31%
13	ca ▲	1,140	18.18%	tv	744,293	1.46%	it	1,157,194	2.27%
14	eu ▲	1,134	18.08%	it	697,559	1.37%	es	1,147,938	2.25%
15	ac.uk ▲	1,122	17.89%	sa	689,872	1.35%	ru	827,668	1.62%
16	st	1,022	16.30%	co.uk	523,268	1.02%	to	781,909	1.53%
17	to	957	15.26%	co.jp	501,987	0.98%	tv	744,293	1.46%
18	info	949	15.13%	to	415,100	0.81%	sa	689,872	1.35%
19	es	896	14.29%	nu	337,159	0.66%	gd	584,337	1.14%
20	tv	888	14.16%	ms	317,668	0.62%	co.uk	523,268	1.02%

The TLDs are ordered by the number of users (#users) which have posted a URL with the corresponding TLD in one of their tweets. The third column block shows the counts for the original sample data without removing shortened URLs. The highlighted TLDs ▲ in the computer scientists data do not appear among the top 20 of the sample.

since their host name is not among the top 10,000 MAG publisher hosts. For this table we have used a threshold of 20, that is, only URLs which have been shared by more than 20 users in the sample are included. We observed that the larger threshold provided a better balance between relevance for the computer scientists and the general relevance on Twitter in this case, where the URLs also have been frequently tweeted by the sample users. By some margin the highest ranked URL is the blog post from Twitter, announcing their data grants that allow selected researchers access to the complete Twitter data. This is also the topic of the 4th URL. Upon inspection, the remaining URLs are also clearly relevant for computer scientists, e.g., about the visualization of algorithms (2), git manuals (3), comics about challenges in AI, thesis defense, programming languages, and academic Twitter use (5, 6, 13, and 18), data analysis (7), history of cryptography (8), the passing of the Turing test (9), HTML5 (11), security of git clients (14), proliferation of apps (15), programming languages (16), computer graphics (17), AI/neural networks (19) and a Taylor Swift parody on online security (20). The PhD Comic (18) is somewhat special because it actually cites and transforms a Nature article from 2014 on the use of Twitter by scientists, which is also on the list of top publications (see Section 3.6). It is apparent that most links point to websites that post relevant content for computer scientists and have some degree of entertainment value as well.

Table C. The top 20 domains ordered by the odds ratio.

domain	OR_{lb}	OR	OR_{ub}	$\#u_{CS}$	$\#u_S$
lemire.me	22,284	52,647	124,380	108	17
videolectures.net	18,502	51,518	143,452	75	12
computer.org	23,344	44,520	84,905	165	31
johndcook.com	18,819	40,682	87,946	108	22
acm.org	31,779	40,306	51,122	1023	247
socialmediacollective.org	15,369	38,117	94,536	74	16
regehr.org	12,042	32,279	86,526	55	14
yhathq.com	12,241	31,785	82,535	58	15
scikit-learn.org	12,429	31,355	79,099	61	16
strataconf.com	14,399	29,893	62,057	94	26
datasociety.net	10,569	29,667	83,278	47	13
academictorrents.com	12,699	29,243	67,344	71	20
insidehpc.com	12,501	28,827	66,472	70	20
pyimagesearch.com	9,241	27,322	80,777	40	12
the-paper-trail.org	11,313	26,842	63,684	62	19
usenix.org	16,954	26,520	41,482	226	72
toronto.edu	10,439	26,095	65,231	54	17
might.net	10,536	24,669	57,760	60	20
continuum.io	8,901	24,598	67,974	42	14
epsrc.ac.uk	8,562	24,586	70,595	39	13

The table extends the domain data from Table 5 with the lower (OR_{lb}) and upper (OR_{ub}) bounds for the 99.9% confidence intervals of the odds ratio.

F Sample Tweets for some of the Publications from Table 7

For ethical reasons, user names of Twitter users were removed and replaced by generic user name (i.e., @A, @B, . . .).

1 ◇ Repeatability and Benefaction in Computer Systems Research. ***Collberg, Proebsting, Warren*** This paper received both many retweets and original tweets. There are also tweets which critically deal with the paper and its results, for instance:

- "SIGIR papers weren't examined in this study but one wonders",
- "A study naming CS authors who withheld their research data. Valid point, but Is it ethical? Did the authors consent?", or
- "Slightly ironic if their research can't be replicated for ethical reasons".

2 △ Genes mirror geography within Europe. ***Novembre et al.***

- The tweet "Incredible, running PCA on the genes of 3,000 Europeans gives you a map of Europe http://t.co/1cd9o7IkBa http://t.co/2Rrpj9hS8w" (on February 23, 2014, at 11:23) is retweeted 29 times.
- The tweet "This is just too cool! PCA applied to Europeans' genes reproduces geographical map of Europe http://t.co/JNao2DbSPK http://t.co/5FE1EOZog5" (on February 25, 2014, at 13:25) is retweeted 2 times.

Table D. The top 20 hosts ordered by the odds ratio.

host	OR_{lb}	OR	OR_{ub}	$\#u_{CS}$	$\#u_{S}$
yahoolabs.tumblr.com	34,932	79,062	178,939	170	18
dl.acm.org	36,254	56,710	88,710	410	63
lemire.me	22,284	52,647	124,380	108	17
videolectures.net	17,453	50,899	148,439	68	11
cacm.acm.org	30,050	48,131	77,089	325	58
www.computer.org	22,803	46,650	95,436	140	25
www.johndcook.com	20,063	44,750	99,815	108	20
stanford.edu	15,997	40,658	103,340	74	15
nlp.stanford.edu	16,543	39,805	95,776	82	17
socialmediacollective.org	15,369	38,117	94,536	74	16
www.cs.cmu.edu	21,759	37,094	63,237	207	47
colah.github.io	10,810	32,807	99,567	44	11
agenda.weforum.org	13,620	32,493	77,517	71	18
blog.regehr.org	11,799	31,687	85,097	54	14
scikit-learn.org	12,429	31,355	79,099	61	16
homepages.inf.ed.ac.uk	11,556	31,095	83,667	53	14
cs.stanford.edu	16,009	30,872	59,534	119	32
homes.cs.washington.edu	11,774	30,679	79,941	56	15
strataconf.com	14,399	29,893	62,057	94	26
www.datasociety.net	10,569	29,667	83,278	47	13

The table extends the host data from Table 5 with the lower (OR_{lb}) and upper (OR_{ub}) bounds for the 99% confidence intervals of the odds ratio.

- On February 25, 2014, there are 6 further retweets: “RT @A: MT @B: PCA on the genes of 3,000 Europeans gives map of Europe http://t.co/mpUdE3MiCI”.

3 □ Publishers withdraw more than 120 gibberish papers. ***van Noorden***
The paper received some retweets but also many retweets which critically deal with the topic.

11 △ Rotational Splittings with CoRoT, Expected Number of Detections and Measurement Accuracy. ***Goupil, Lochard, Samadi, Barban, Dupret, Baglin***

- There are retweets of a tweet of a user which itself is not contained in our dataset which appeared in April (6) and also May (1) and June (3). An example of a retweet from April 4, 2014, at 2:14 is “RT @C: First-known modern example of an ANTI-acknowledgment in a serious technical paper. http://t.co/aYACPVS7eF http://t.co/UGNx9MQ. . . ”.
- Another user not contained in our dataset picks this up and is retweeted in June (10) but also in July (1), October (5) and November (2). The first reweet is from June 10, 2014, at 15:24: “RT @D: The ”anti-acknowledgement” section. via @C http://t.co/AsQ1UEcjRN http://t.co/h8drp4BVZr”.
- On June 7, 2014, another user comments: “What we often wish we could say in #academia.. http://t.co/dQxGPxntxb http://t.co/zP3W4d3r7w”.

Table E. The top 20 publisher domains ordered by the odds ratio.

domain	OR_{lb}	OR	OR_{ub}	$\#u_{CS}$	$\#u_{S}$
ceur-ws.org	39,320.3	156,199	620,498.1	113	6
aaai.org	23,630.7	71,015	213,414.2	86	10
nott.ac.uk	15,780.7	65,657	273,171.6	48	6
umontreal.ca	19,405.8	56,202	162,766.8	75	11
umd.edu	23,206.8	53,475	123,220.3	116	18
vldb.org	11,153.5	47,775	204,640.6	35	6
computer.org	23,344.2	44,520	84,905.2	165	31
arizona.edu	16,433.5	42,967	112,341.2	73	14
acm.org	31,779.2	40,306	51,121.6	1023	247
aclweb.org	12,828.4	40,221	126,107.4	49	10
gla.ac.uk	9,859.8	35,831	130,214.0	35	8
ucsb.edu	7,978.1	35,439	157,420.8	26	6
utah.edu	8,805.5	35,072	139,690.5	30	7
toronto.edu	11,635.0	35,061	105,654.6	47	11
cmu.edu	21,338.6	32,943	50,857.9	282	73
tue.nl	7,807.6	31,550	127,488.3	27	7
soton.ac.uk	8,278.7	30,688	113,756.4	30	8
cornell.edu	13,880.8	30,148	65,480.2	84	23
ucdavis.edu	7,144.7	29,203	119,365.1	25	7
sigcomm.org	6,230.3	28,601	131,294.4	21	6

The table extends the domain data from Table 6 with the lower (OR_{lb}) and upper (OR_{ub}) bounds for the 99% confidence intervals of the odds ratio.

12 ○ Links that speak: the global language network and its association with global fame. ***Ronen, Goncalves, Hu, Vespignani, Pinker, Hidalgo*** The tweets mainly promote the paper or copy its title/teaser.

14 □ The missing piece to changing the university culture. ***Schillebeeckx, Maricque, Lewis*** The paper received mainly retweets.

18 □ The rise and rise of citation analysis. ***Meho*** Of the 12 tweets the paper received, 9 are retweets of a user not contained in our dataset which appeared at the end of March 2014. An example is this tweet from March 24, 2014, at 9:34: "RT @E: 90% of papers published in academic journals are never cited; 50% never read by anyone but author, editor & reviewers h. . . ". Three further tweets have an almost identical wording.

19 ◇ An Updated Performance Comparison of Virtual Machines and Linux Containers. ***Felter, Ferreira, Rajamony, Rubio*** Overall, the paper received rather few tweets which mainly copy the title. Interesting is a tweet saying "Looks like IBM JUST discovered what we in #illumos and #solaris knew for 10y.".

a □ Online collaboration: Scientists and the social network. ***van Noorden*** The paper received mainly retweets, some of them critical. The corresponding PhD comic is sometimes tweeted alongside.

d ◇ Deep Learning. ***Bengio, Goodfellow, Courville*** The paper received many retweets, most of them in appreciation of the new book.

Table F. The top publications from the computer scientists dataset.

	publication	year	#cit	$\#u_{CS}$	$\#u_S$	OR_{lb}	OR	OR_{ub}
1	◇ Repeatability and benefaction in computer systems research. *Collberg, Proebsting, Warren*	2014	5	69	6	23,321	94,702	384,559
2	△ Genes mirror geography within Europe. *Novembre et al.*	2008	720	45	10	11,673	36,914	116,734
3	□ Publishers withdraw more than 120 gibberish papers. *van Noorden*	2014	44	**118**	27	17,954	36,276	73,296
4	◇ Python is now the most popular introductory teaching language at top U.S. universities. *Guo*	2014	19	76	19	14,163	32,977	76,782
5	◇ Interactive notebooks: Sharing the code. *Shen*	2014	29	28	7	8,140	32,723	131,554
6	◇ Deep neural networks are easily fooled: High confidence predictions for unrecognizable images. *Nguyen, Yosinski, Clune*	2014	98	45	12	10,551	30,762	89,686
7	◇ Please put OpenSSL out of its misery. *Kamp*	2014	4	26	8	7,021	26,579	100,619
8	□ An efficiency comparison of document preparation systems used in academic research and development. *Knauff, Nejasmic*	2014	1	57	19	10,301	24,657	59,020
9	◇ The network is reliable. *Bailis, Kingsbury*	2014	16	17	6	4,846	23,138	110,476
10	□ Publishing: The peer-review scam. *Ferguson, Marcus, Oransky*	2014	36	24	9	6,019	21,802	78,965
11	△ Rotational splittings with CoRoT, expected number of detections and measurement accuracy. *Goupil, Lochard, Samadi, Barban, Dupret, Baglin*	2006	1	28	11	6,451	20,824	67,217
12	○ Links that speak: The global language network and its association with global fame. *Ronen, Goncalves, Hu, Vespignani, Pinker, Hidalgo*	2014	27	24	11	5,378	17,838	59,164
13	◇ To wash it all away. *Mickens*	2014	0	20	10	4,565	16,341	58,495
14	□ The missing piece to changing the university culture. *Schillebeeckx, Maricque, Lewis*	2013	29	25	13	5,100	15,725	48,489
15	□ Scientific method: Statistical errors. *Nuzzo*	2014	170	**96**	58	7,907	13,690	23,702
16	○ Experimental evidence of massive-scale emotional contagion through social networks. *Kramer, Guillory, Hancock*	2014	422	**45**	28	5,964	13,184	29,143
17	□ Lectures aren't just boring, they're ineffective, too, study finds. *Bajak*	2014	4	26	17	4,477	12,508	34,942
18	□ The rise and rise of citation analysis. *Meho*	2007	227	12	8	2,724	12,240	55,004
19	◇ An updated performance comparison of virtual machines and linux containers. *Felter, Ferreira, Rajamony, Rubio*	2014	67	9	6	2,158	12,234	69,354
20	○ Trolls just want to have fun. *Buckels, Trapnell, Paulhus*	2014	89	9	6	2,158	12,234	69,354
a	□ Online collaboration: Scientists and the social network. *van Noorden*	2014	85	**79**	70	5,415	9,309	16,003
b	△ Variation in melanism and female preference in proximate but ecologically distinct environments. *Culumber et al.*	2014	3	**73**	63	5,413	9,548	16,841
c	□ Nature promotes read-only sharing by subscribers. *van Noorden*	2014	2	**63**	56	5,050	9,255	16,963
d	◇ Deep learning. *Bengio, Goodfellow, Courville*	2014	71	47	3	18,105	128,558	912,861
e	○ Big data, hype, the media and other provocative words to put in a title. *Jordan*	2014	0	44	4	16,169	90,220	503,423
f	◇ First-person hyper-lapse videos. *Kopf, Cohen, Szeliski*	2014	37	**43**	40	4,273	8,816	18,186
g	◇ Computer science: The learning machines. *Jones*	2014	0	40	4	14,585	81,966	460,642
h	□ How to build a bad research center. *Patterson*	2014	0	40	0	-	∞	-
i	◇ Do we need hundreds of classifiers to solve real world classification problems?. *Fernández-Delgado et al.*	2014	152	35	2	13,093	143,325	1,568,905
j	□ The top 100 papers. *van Noorden, Maher, Nuzzo*	2014	72	**32**	26	4,221	10,392	24,047
k	◇ Extracting audio from visual information. *Hardesty*	2014	1	**32**	37	3,195	7,080	15,687

The table extends the publication data from Table 7 with the lower (OR_{lb}) and upper (OR_{ub}) bounds for the 99% confidence intervals of the odds ratio.

Table G. The top 20 URLs from hosts that are not among the top 10,000 of the MAG publisher host list.

OR_{lb}	OR	OR_{ub}	$\#u_{CS}$	$\#u_{S}$	URL
11,693	22,446	43,086	95	35	https://blog.twitter.com/2014/introducing-twitter-data-grants
7,974	13,979	24,505	93	55	http://bost.ocks.org/mike/algorithms/
5,317	13,030	31,932	35	22	http://git-man-page-generator.lokaltog.net/
5,498	12,610	28,922	40	26	http://www.scientificamerican.com/article/twitter-to-release-all-tweets-to-scientists-a-trove-of-billions-of-tweets-will-be-a-research-boon-and-an-ethical-dilemma/
5,803	12,441	26,672	47	31	http://xkcd.com/1403/
6,052	11,831	23,131	59	41	http://xkcd.com/1425/
5,806	11,766	23,842	53	37	https://jawbone.com/blog/napa-earthquake-effect-on-sleep/
4,820	11,601	27,923	34	24	http://www.telegraph.co.uk/history/world-war-two/10810980/Female-codebreakers-reunited-at-Bletchley-Park.html
4,426	11,159	28,134	30	22	https://www.techdirt.com/articles/20140609/07284327524/no-supercomputer-did-not-pass-turing-test-first-time-everyone-should-know-better.shtml
4,283	10,674	26,599	30	23	http://blog.okcupid.com/index.php/we-experiment-on-human-beings/
4,848	10,435	22,464	42	33	http://www.w3.org/blog/news/archives/4167
4,602	9,886	21,234	41	34	http://bjorn.tipling.com/if-programming-languages-were-weapons
4,383	9,778	21,814	37	31	http://www.npr.org/blogs/money/2014/10/17/356944145/episode-576-when-women-stopped-coding
4,626	9,435	19,243	46	40	https://github.com/blog/1938-vulnerability-announced-update-your-git-clients
3,547	9,292	24,339	25	22	http://www.codinghorror.com/blog/2014/02/app-pocalypse-now.html
3,547	9,292	24,339	25	22	http://hacklang.org/
3,794	9,093	21,791	30	27	http://www.cgsociety.org/index.php/CGSFeatures/CGSFeatureSpecial/building_3d_with_ikea
3,431	8,888	23,022	25	23	http://www.phdcomics.com/comics.php?f=1737
3,592	8,810	21,611	28	26	http://www.i-programmer.info/news/105-artificial-intelligence/7985-a-worms-mind-in-a-lego-body.html
3,200	8,546	22,823	23	22	http://swiftonsecurity.tumblr.com/post/98675308034/a-story-about-jessica

The URLs are ranked by their odds ratio (OR). Only URLs which have been shared by more than 20 users in the sample dataset are included.
